# The Sphygmograph

**Published:** 1874-04

**Authors:** 


*The Sphygmograph: Its Physiological and Pathological Indications, with Illustrations.* By Edgar Holden, A. M., M. D. Philadelphia: Lindsay & Blakiston, 1874.

We presented our readers in the January number of this Journal, a description of Dr. Holden's Sphygmograph, together with an engraving of the instrument. The author claims that many of the disappointments which have arisen in the application of the Sphygmograph are due to faults in the construction of the instrument which he has over come in the one which he has invented and describes in this monograph. Part first is devoted to an explanation of the mechanism of the Sphygmograph and its action. Part second describes its practical application, and part third the results of observations made concerning the actions of various remedies upon the pulse.

The whole subject is well considered and is a clear statement of the advantages which may be obtained in the use of the sphygmograph. We do not however consider that it will ever come into use in general practice, it may be of value in certain observations to be made at the bed-side, but it is only in hospital practice that it can be utilized. Dr. Holden's observations on the modification of the pulse by certain drugs are well made and carefully recorded; his essay is a valuable contribution to scientific medicine.

